# Spectrum of imaging findings in AIDS-related diffuse large B cell lymphoma

**DOI:** 10.1186/s13244-020-00871-w

**Published:** 2020-05-19

**Authors:** Edward Chege Nganga, Samuel Gitau

**Affiliations:** Aga Khan University Hospital Nairobi, 3rd Parklands Avenue, Limuru Road, Nairobi, Kenya

**Keywords:** AIDS-related diffuse large B cell lymphoma, Multimodality imaging of lymphoma, Extranodal lymphoma, HIV and malignancy

## Abstract

Lymphoma in HIV-infected patients is AIDS defining. This is the second most common AIDS defining malignancy after Kaposi’s sarcoma. Development of lymphoma in HIV patients is related to immunosuppression and high viral load. Co-infection with other lymphotrophic viruses especially EBV is also strongly associated with development of lymphoma in HIV patients. Despite advances in HAART therapy, incidence of diffuse large B cell lymphoma in HIV-infected patients remains significantly higher than in the general population.

Early diagnosis is challenging due to presence of opportunistic infections and atypical presentation of the lymphoma in this subset of patients. Atypical imaging findings are not unusual, and the diagnosis of lymphoma on imaging is on many occasions unexpected as the patient would ideally be initially investigated for presumed opportunistic infection.

Lymphoma treatment approaches in HIV patients are complicated by comorbidity with opportunistic infections and performance status of the patients. Treatment failure and early relapse are also common in AIDS-related lymphoma. This review article highlights the common and unusual multimodality imaging findings in HIV-associated lymphoma.

## Teaching points

- Non-Hodgkin lymphoma is the second most common malignancy in HIV patients.
- AIDS-related NHL is an aggressive multisystem disease with a poor prognosis.
- CT and FDG PET CT imaging are crucial in initial diagnosis, staging, and follow-up of AIDS-related lymphoma.
- Knowledge of imaging features of diffuse features of HIV-associated lymphoma can help to differentiate lymphoma from infectious comorbidities which are common in HIV/AIDS patients.

## Introduction

HIV remains a critical health problem; as of 2018, 37.9 million people globally were living with HIV, of which 24.5 million had access to highly active antiretroviral therapy (HAART) [1]. In 2018, approximately 770,000 people died from AIDS-related illnesses. This represents a significant reduction by more than 56% since the peak in 2004. UNAIDS estimates that approximately $26.2 billion will be required worldwide for the AIDS response in 2020 [1]. As the lifespan of HIV-infected patients is increasing, malignancies now contribute a significant cause of morbidity and mortality in HIV-infected patients. Non-Hodgkin’s lymphoma is the second most common HIV-related malignancy after Kaposi’s sarcoma [2]. The other AIDS defining malignancy is invasive cervical cancer [3].

Twenty-five to 40% of HIV-positive patients will develop a malignancy; approximately 10% will be non-Hodgkin’s lymphoma. Since implementation of ART, the risk of non-Hodgkin’s lymphoma initially declined, likely related to improvement in CD4 counts [4, 5]. The risk of developing lymphoma remains higher in those with low CD4 counts and high HIV viral loads [6]. Approximately 75% of the non-Hodgkin’s lymphoma in HIV patients is aggressive diffuse large B cell lymphoma (DLBCL). Indolent lymphomas comprise less than 10% of HIV-related lymphomas [2, 5].

The pathogenesis of non-Hodgkin’s lymphoma in HIV is not well understood. Epstein-Barr virus is however known to play an important role [7]. HIV lymphomas are commonly derived from B cells. Immune dysregulation also plays an important role, with progression of lymphoma associated with interleukin 6 and interleukin 10 production. Genetic dysregulation is also a prominent feature in AIDS-related diffuse large B cell lymphoma, notably BCL 6 expression, and *Myc* overexpression. BCL-2 activation is generally not seen in HIV-related lymphoma.

### Imaging in HIV and lymphoma

The imaging findings of NHLs are primarily masses and lymphadenopathy [8, 9]. Extranodal DLBCL is common in the setting of HIV. Most HIV-positive patients have concomitant infectious disease, or are initially evaluated for presumed infectious disease. Initial imaging prior to diagnosis is guided by clinical presentation and index of clinical suspicion.

The primary imaging modality is usually contrast-enhanced CT. Besides the established imaging findings of nodal enlargement in HIV negative patients, other unusual findings are not uncommon in AIDS-related lymphoma. These include necrosis of the involved nodes, large extranodal soft tissue masses, and bone destruction secondary to lymphoma involvement. AIDS-related lymphoma also presents with multi-organ involvement and bulky disease [10]. 18 FDG PET/CT is a useful tool in initial staging and response assessment of lymphoma in both HIV-positive and HIV-negative patients [11, 12]. The high-grade HIV-associated DLBCLs are more likely to show avid uptake of 18 F-FDG. It is also useful in assessment of sites of FDG avid disease which may not be immediately apparent on conventional CT. PET CT is however inherently limited in tissues which show high background FDG uptake such as the brain.

#### Lung findings

**Fig. 1:**
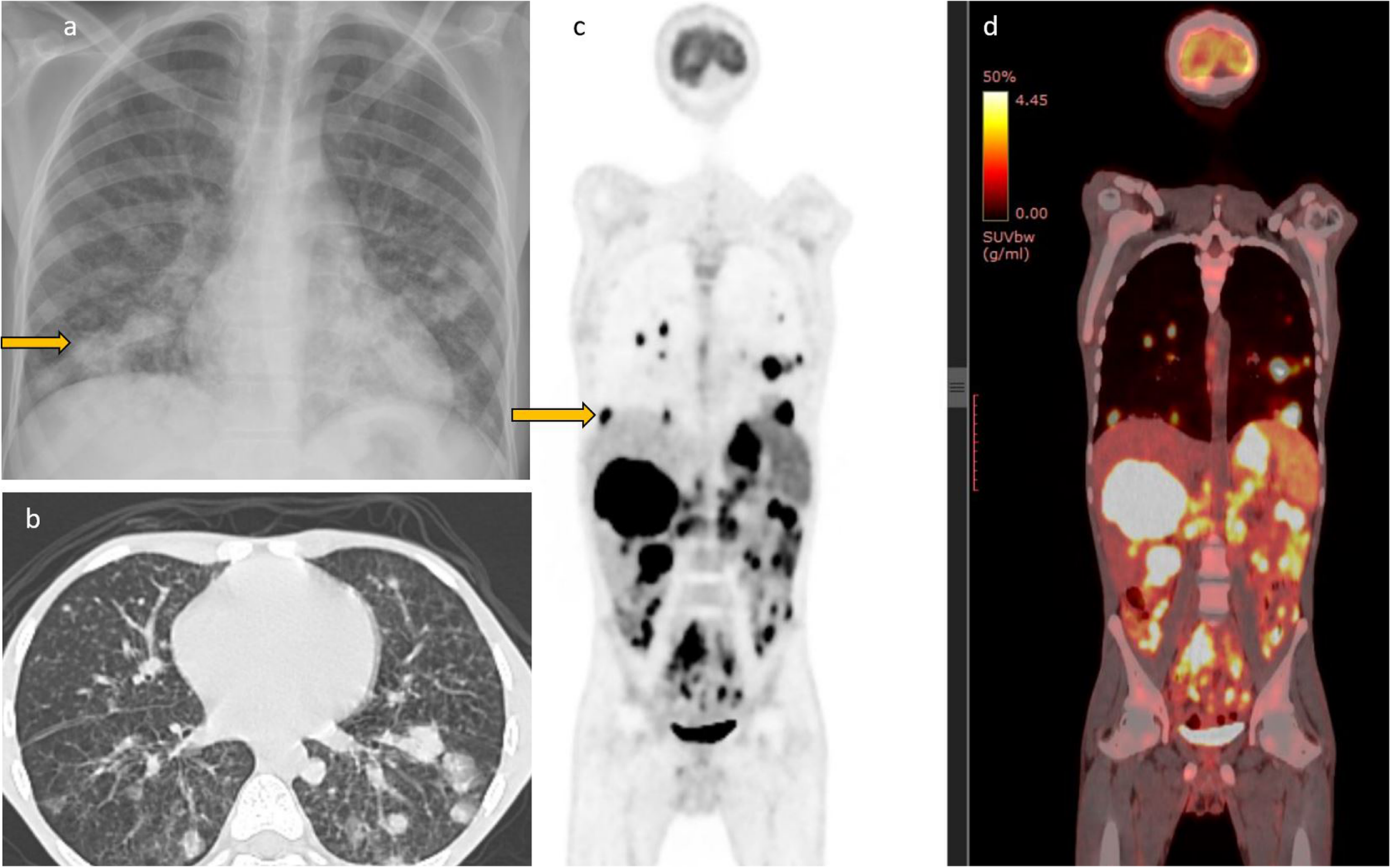
Chest radiograph of a 19-year-old HIV-positive patient with low CD4 count. He presented with cough and dyspnea and was initially being evaluated for possible atypical pneumonia. The chest radiograph showed multiple focal opacities predominantly in the lower lung zones. On CT, there were lung parenchymal nodules/masses of variable sizes which were also intensely FDG avid on PET CT. There was extensive lymphoma involvement elsewhere including in the liver, spleen (splenic activity higher than liver background), and multiple nodal sites above and below the diaphragm. Biopsy of the liver lesion was consistent with DLBCL

Pulmonary involvement of diffuse large B cell lymphoma is usually a manifestation of systemic disease rather than primary pulmonary lymphoma [13]. A few isolated case reports of primary pulmonary lymphoma in the setting of HIV have been described. Up to two-thirds of patients present with respiratory symptoms of cough and dyspnea and would therefore be evaluated for an infective process, initially with the chest radiograph and CT chest. On imaging, pulmonary lymphoma can appear as an isolated mass, multiple pulmonary nodules/masses, or diffuse consolidative opacities [14] (Fig. 1). High-grade pulmonary lymphoma in HIV is invariably 18 FDG avid. Patients may also present with pleural effusion.

#### Gastrointestinal tract findings

**Fig. 2:**
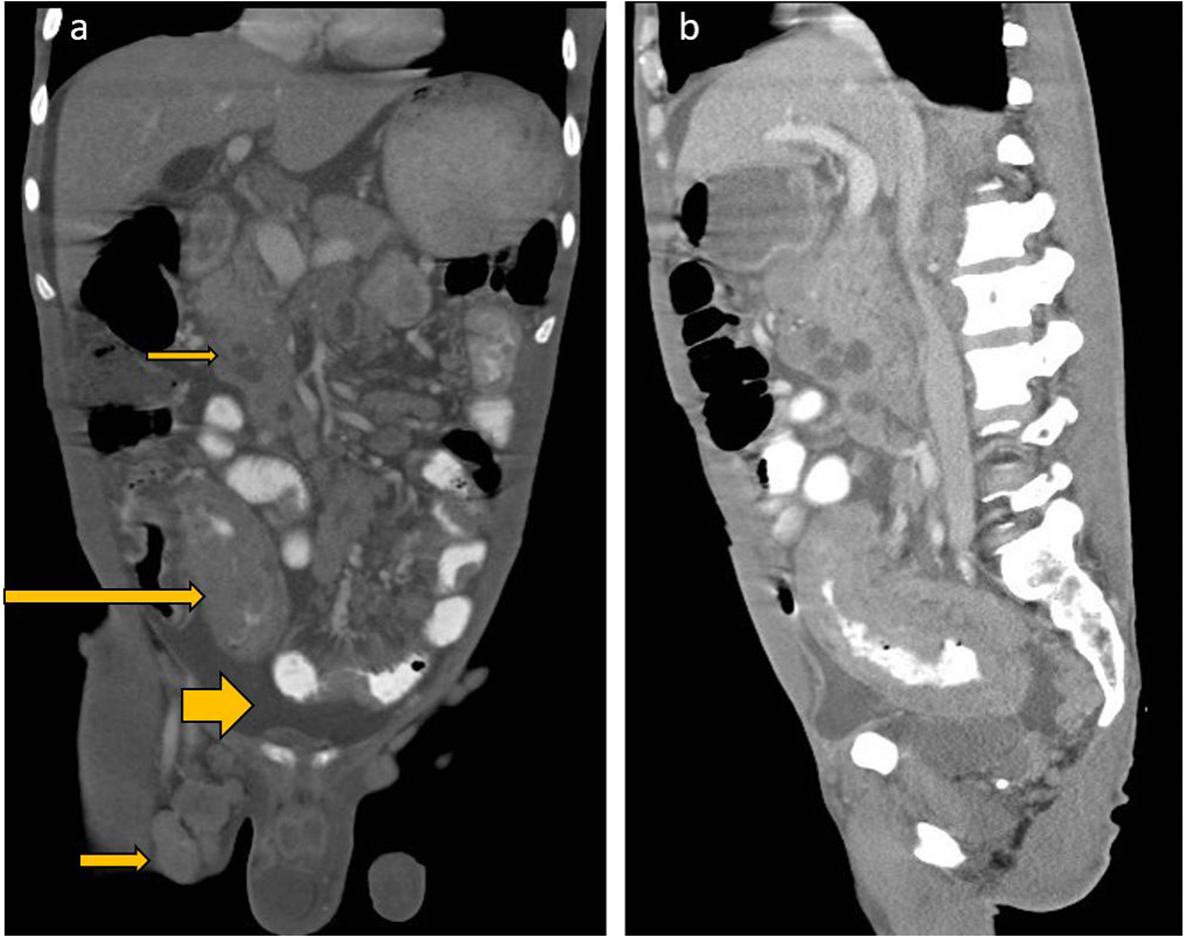
Coronal reformats of a CECT abdomen and pelvis of a recently diagnosed HIV-positive patient who presented with abdominal pain, diarrhea, and weight loss show long-segment bowel thickening involving the distal ileum and caecum (long arrow). There was also mesenteric lymphadenopathy, with some necrotic nodes, (short slender arrow) and a moderate ascites (short fat arrow). There were also enlarged centrally necrotic right inguinal lymph nodes

Gastrointestinal tract is the most commonly affected extranodal site in AIDS-related NHL and is usually multifocal. The symptoms depend on the site of involvement and range from abdominal pain, diarrhea weight loss, gastrointestinal bleeding, or symptoms related to perforation. In a small series of 48 patients, the stomach and duodenum were the most commonly involved site. Bowel involvement presents as soft tissue bowel thickening, typically with associated dilatation rather than narrowing of the bowel lumen (Fig. 2), due to involvement of the myenteric plexus [15]. There is usually associated mesenteric lymphadenopathy. Some patients may also present with ascites.

#### Liver and spleen

**Fig. 3:**
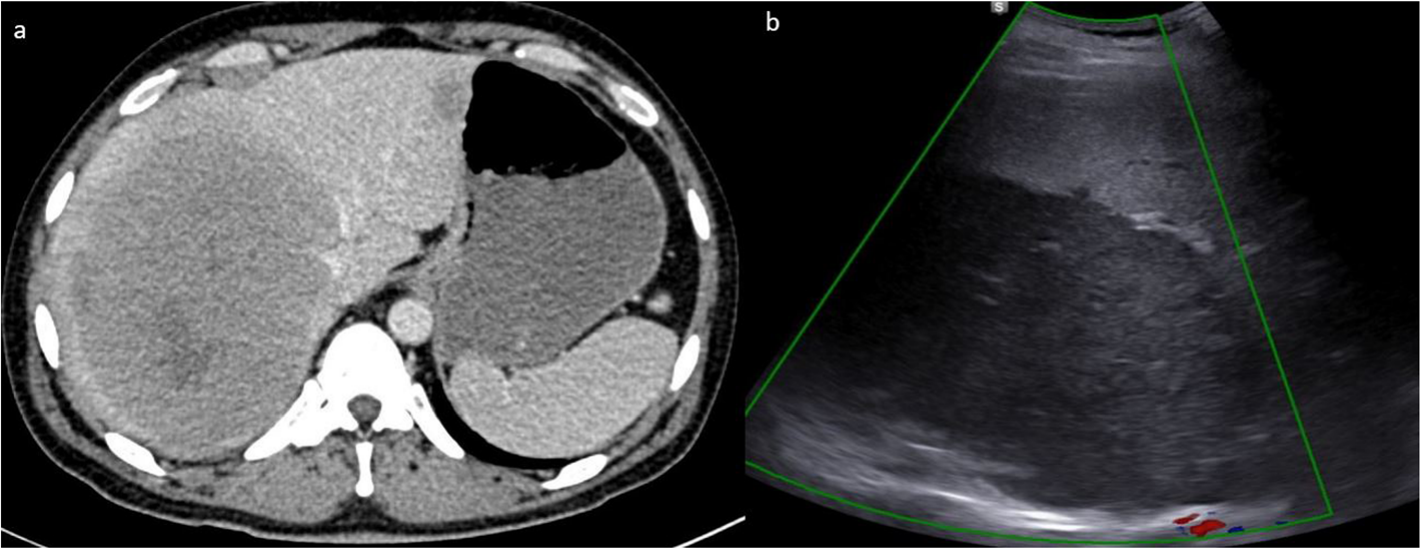
Axial CT images axial contrast-enhanced CT image and liver ultrasound of a patient with HIV-associated primary hepatic lymphoma. The contrast-enhanced CT shows a homogeneous soft tissue attenuation mass which does not show significant enhancement, and with fear preservation of the surrounding liver architecture. The mass displaces or encases the hepatic veins but does not invade vasculature or lead to tumor thrombus. On ultrasound, it appears as a fairly homogenous mass hypoechoic to the liver parenchyma. There is no increase vascularity within the mass or in its periphery

**Fig. 4:**
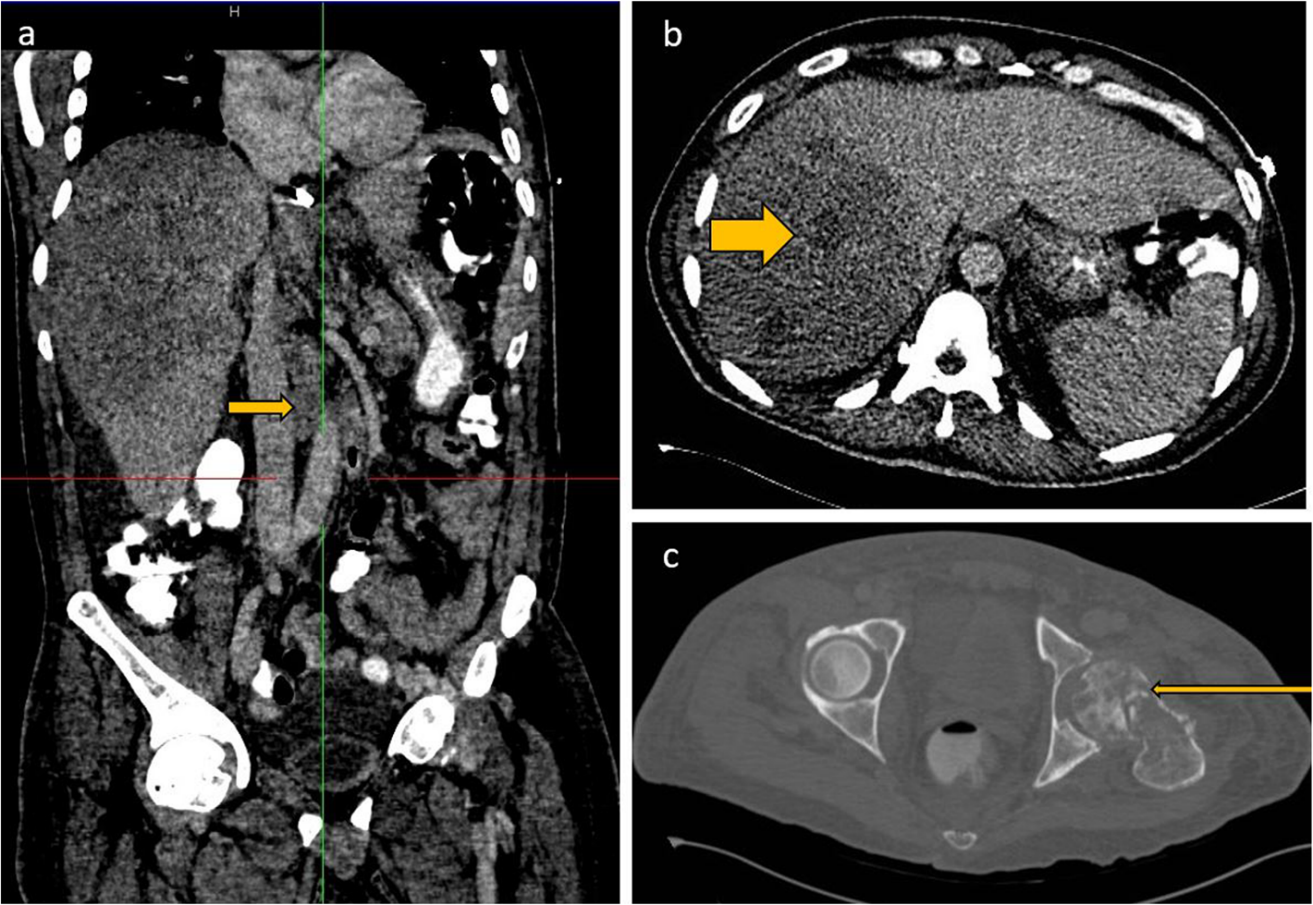
Coronal and axial CECT images of the abdomen (**a** and **b**) show hepatomegaly and a heterogenous hypoattenuating mass infiltrating through most of the right lobe of the liver. The infiltrative liver mass had more focal hypoattenuating areas (fat arrow). Aortocaval lymph nodes are also noted (short arrow). Axial CT through the pelvis in bone window (**c**) shows a permeative process involving the left femoral head and neck and associated pathological fracture

Liver and spleen involvement are not uncommon in AIDS-related lymphoma. This can either be focal or diffuse involvement, with hepatosplenomegaly and focal masses on imaging. These appear as hypoattenuating focal lesions which do not show any significant enhancement on all phases. Additionally, the masses typically encase but do not invade the hepatic vasculature or biliary tree [16]. Absence of significant arterial phase enhancement, vessel penetration sign, and absence of vascular thrombosis are useful clues in diagnosing lymphoma [17] (Fig. 3). In severely immunocompromised lesions, the hypoattenuating focal lesions may demonstrate ring enhancement on contrast-enhanced CT and can easily be confused with tuberculous or fungal microabscesses (Figs. 3 and 4). FDG PET CT may demonstrate avid focal deposits within these organs. In the absence of a focal lesion, diffusely increased FDG uptake within the spleen, higher than the liver background, is considered diffuse involvement of the spleen (Fig. 1). It should be noted that diffuse splenic FDG uptake can also be reactive. This is especially important in patients on chemotherapy.

#### Other solid organs

**Fig. 5:**
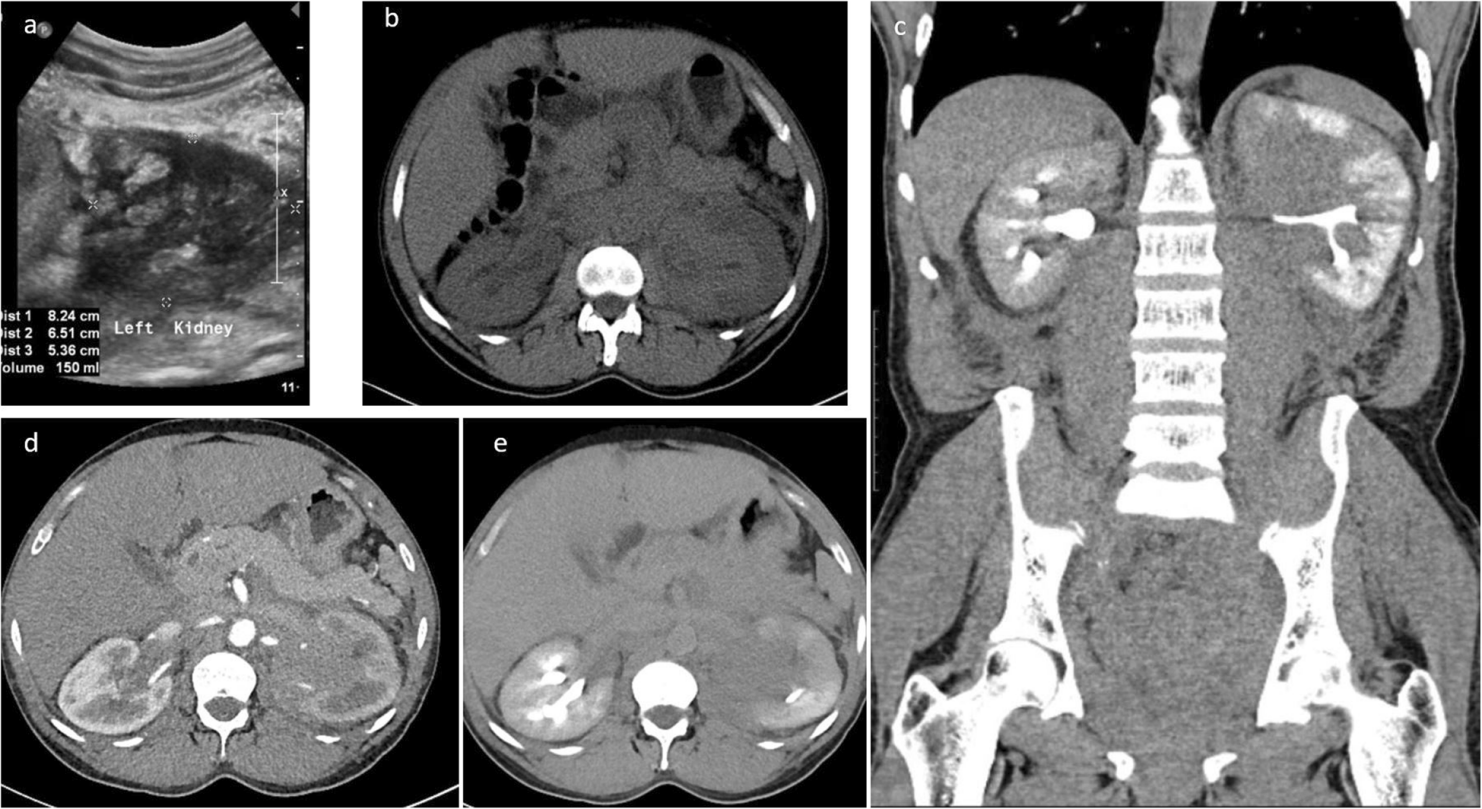
Ultrasound image of the left kidney in a HIV-positive male demonstrates heterogeneously echogenic areas in the medulla extending to the collecting system. Unenhanced CT KUB showed diffuse thickening of the renal pelvis with hyperdense soft tissue material relative to the renal parenchyma. Heterogenous infiltration of the kidney is seen on the postcontrast CT images in arterial and urographic phases

HIV-associated lymphoma can rarely involve the kidneys, pancreas, and adrenal glands. The incidence of pancreatic involvement has been reported to be up to 5% in HIV patients [18]. The kidneys have no intrinsic lymphoid tissue, and their involvement therefore signifies disseminated disease [8]. On imaging, infiltrative masses tend to be hyperattenuating to the renal parenchyma on the unenhanced CT which can be a useful clue. Lymphomatous infiltration also commonly occurs along the renal capsule and sinus [19] (Fig. 5).

#### Bone findings

**Fig. 6:**
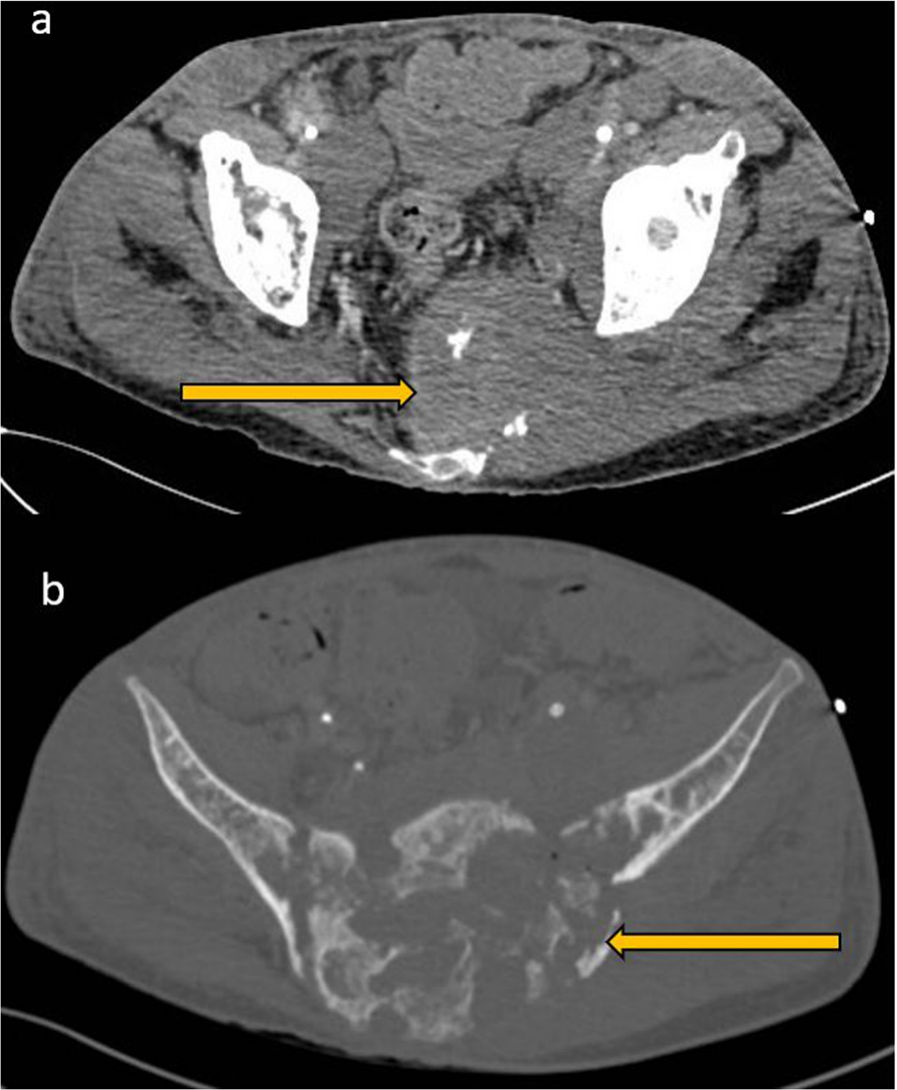
CECT of the pelvis in soft tissue window (**a**) and bone window (**b**). A mixed lytic and sclerotic process involving the sacrum and extending to the iliac bones through the sacroiliac joints is seen. There are associated pathological fractures and a large extra osseous soft tissue component. There are also enlarged necrotic bilateral pelvic sidewall and inguinal lymph nodes

Bone involvement occurs in approximately 5% of disseminated lymphoma cases although this may be higher in HIV. Imaging appearances are nonspecific and range from both mixed lytic and sclerotic lesions and extensive soft tissue involvement [16, 17] (Fig. 6). Evaluation for soft tissue involvement is critical as the isolated bone findings may mimic osteomyelitis. Differential diagnoses also include Ewing’s sarcoma which could present with bone destruction and associated soft tissue component.

### Role of imaging during treatment of HIV-associated DLBCL

Despite advances in HAART and chemotherapy, HIV-associated DLBCL carries a poor prognosis related to the HIV disease and aggressive nature of the lymphoma. The standard therapy is CHOP chemotherapy regimen, with or without rituximab (R-CHOP). Previous studies before rituximab recorded complete response rates of 30–50% and 2-year survival rates of less than 20% [23]. Although rituximab has revolutionized the treatment of CD20-positive lymphomas in the HIV-negative population, its role in HIV-related lymphoma is not as clearly defined, given the profound cellular and humoral immunodeficiency that this agent can cause [24]. Many authors suggest the addition of rituximab to the treatment regimen if the CD4 count is over 50/mL and individualized consideration for patients with CD4 less than 50 [25]. Imaging is crucial in assessing response to treatment. An interim PET/CT is useful in assessment of treatment response.

### Posttreatment surveillance

**Fig. 7:**
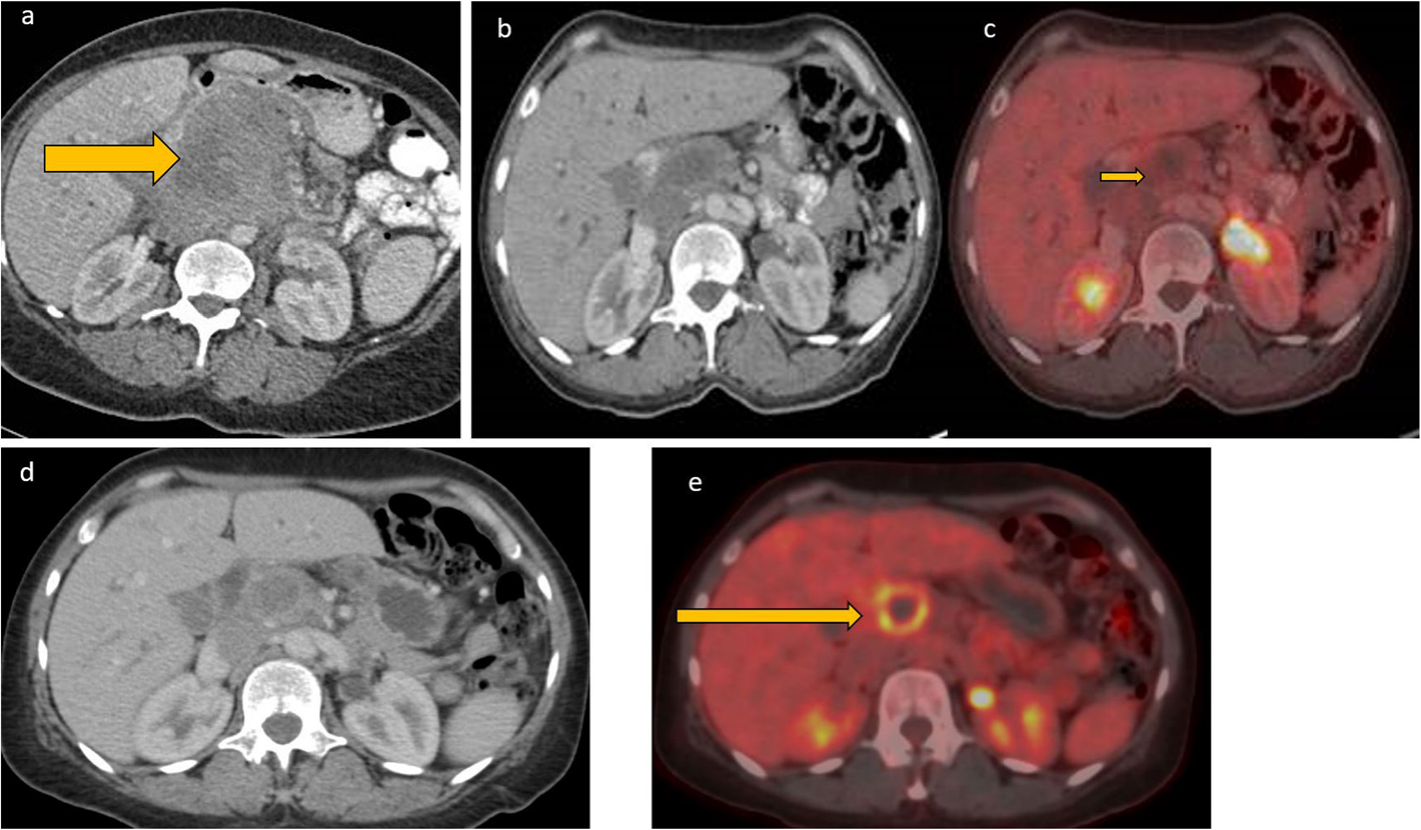
A 46-year-old female patient who had pancreatic lymphoma (**a**) had a negative PET CT at the end of treatment (**b** and **c**). Nine months later, there was local disease recurrence at the pancreatic head on follow-up FDG PET CT scan (**d** and **e**)

**Fig. 8:**
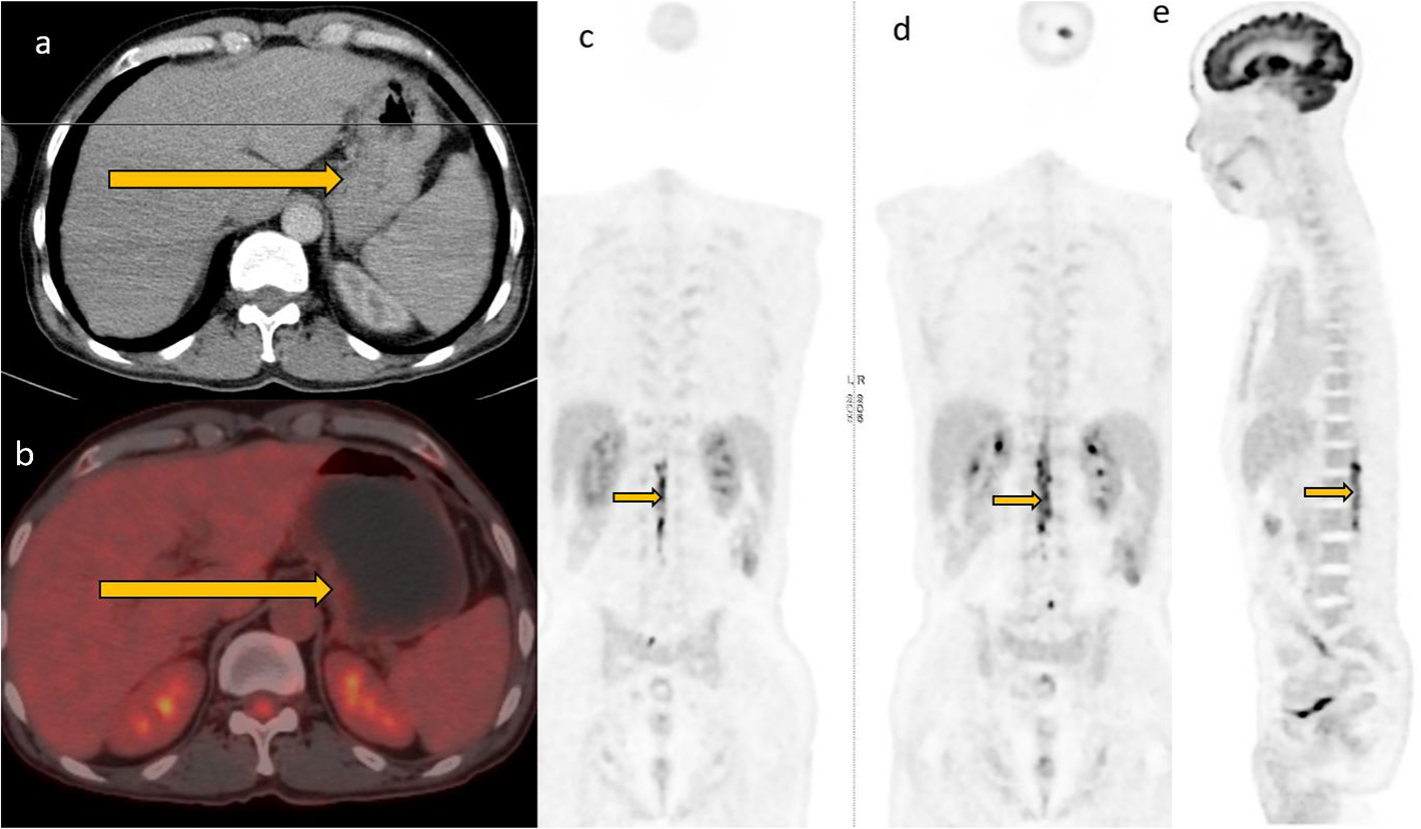
Newly diagnosed HIV-positive patient who had CD4 count of 151 presented with primary gastric lymphoma. He was treated with R-CHOP regimen complicated by severe neutropenic sepsis. Intrathecal chemotherapy was deferred to cycle 3 due to poor performance. Interim PET/CT showed good treatment response of the gastric tumor but there was refractory neuraxial disease seen as FDG avid conus medullaris

**Fig. 9:**
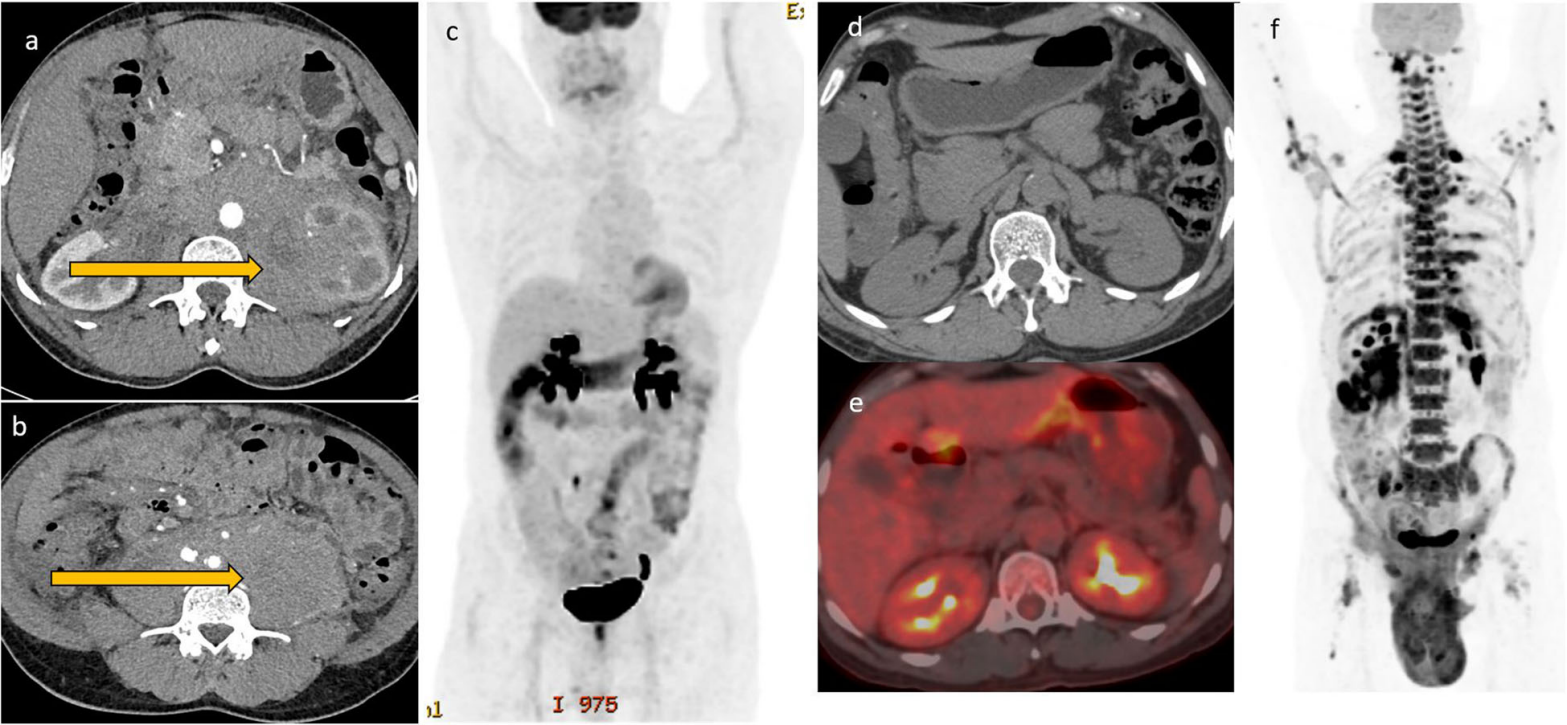
This patient with extensive retroperitoneal and renal disease (**a** and **b**) showed excellent response to treatment in the interim PET CT (**c**, **d**, and **e**) after two cycles of R-CHOP. There was however early relapse with bulky disease developing in the retroperitoneum and the scrotum (**f**)

**Fig. 10:**
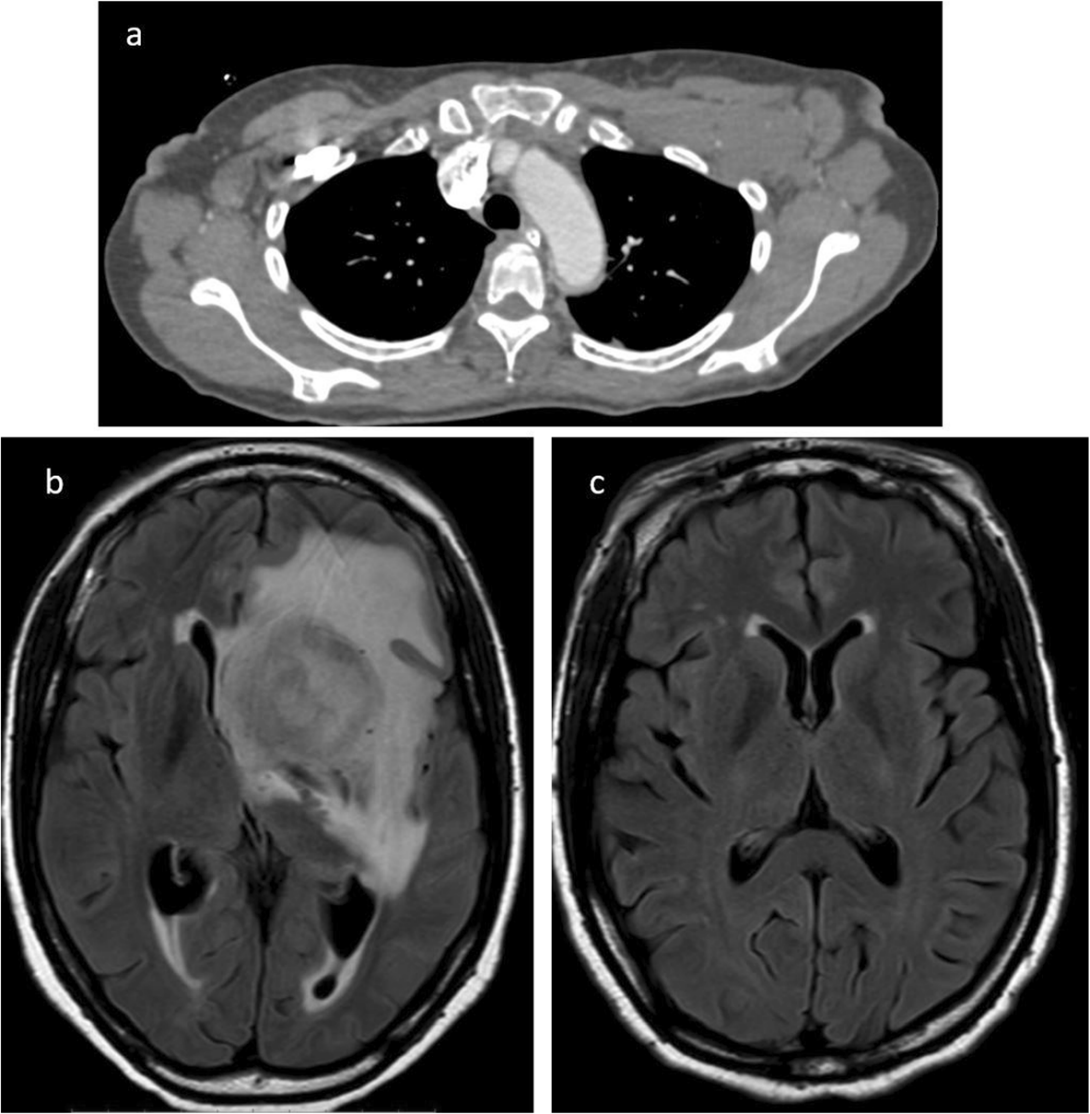
Axial CT image (**a**) demonstrates bilateral axillary lymphadenopathy, proven to be DLBCL after biopsy. The patient successfully completed treatment but developed CNS symptoms 1 year later. MRI of the brain (**b**) showed a large heterogenous mass in the left cerebral hemisphere which biopsy was proven to be lymphoma. A previous MRI brain (**c**) was negative for CNS involvement

Current evidence shows no clinical benefit of surveillance imaging [20, 21], although this is routinely offered to patients in an effort to detect recurrence before the onset of clinical symptoms when patients are more likely to accrue maximal benefit from salvage treatment. In the clinical setting, most patients are evaluated with CT during surveillance. Imaging with PET CT is not recommended for posttreatment surveillance due to high false positive rates. The false positive rates could even be higher in HIV patients due to some opportunistic infections which may have presentation similar to that of lymphoma. Disease recurrence is higher in HIV-positive patients than HIV negative patients. This may be attributable to initial bulk disease, persistent immune dysregulation, and suboptimal treatment in this subset of patients. Recurrence can either be at the site of primary disease or in distant sites. Imaging strategy should therefore be guided by clinical presentation and the degree of clinical suspicion (Figs. 7, 8, 9, and 10).

## Conclusion

Imaging is valuable for diagnosis and differential diagnosis of AIDS-related lymphomas and facilitates staging and monitoring of therapeutic effects. However, there can be atypical imaging features of AIDS-related DLBCL compared to findings in HIV-negative patients. Knowledge of these atypical imaging features may facilitate the diagnosis of lymphoma over infectious disease in the setting of HIV/AIDS.

## Data Availability

Available

## Abbreviations

AIDS: Acquired immunodeficiency syndrome
BCL: B cell Lymphoma
CECT: Contrast-enhanced CT
CT: Computed tomography
DLBCL: Diffuse large B cell lymphoma
EBV: Epstein-Barr virus
FDG: Fluorodeoxyglucose
HAART: Highly active antiretroviral therapy
HIV: Human immunodeficiency virus
NHL: Non-Hodgkin’s lymphoma
PET: Positron emission tomography
R-CHOP: Rituximab, cyclophosphamide doxorubicin, vincristine (Oncovin), and prednisone
